# The association of osteoprotegerin and RANKL with osteoporosis: a systematic review with meta-analysis

**DOI:** 10.1186/s13018-023-04179-5

**Published:** 2023-11-07

**Authors:** Guanghao Chi, Longshun Qiu, Jian Ma, Wei Wu, Yuxin Zhang

**Affiliations:** 1 Department of Orthopedics, Hanzhong Central Hospital, Hanzhong City, 723000 Shanxi Province China; 2 Ping’an Town Health Center Zhenba County, Hanzhong City, 723000 Shanxi Province China; 3 Department of Oral Surgery, Shanghai Ninth People’s Hospital, Shanghai Jiao Tong University School of Medicine, Shanghai, 200011 China; 4 College of Stomatology, Shanghai Jiao Tong University, Shanghai, 200011 China; 5 National Center for Stomatology, National Clinical Research Center for Oral Diseases, Shanghai Key Laboratory of Stomatology, Shanghai Research Institute of Stomatology, Research Unit of Oral and Maxillofacial Regenerative Medicine, Chinese Academy of Medical Sciences, Shanghai, 200011 China; 6 Shanghai Key Laboratory of Orthopedic Implants, Shanghai Ninth People’s Hospital, Shanghai Jiao Tong University School of Medicine, Shanghai, 200011 China; 7 Department of Rehabilitation Medicine, Huangpu Branch, Shanghai Ninth People’s Hospital, Shanghai Jiao Tong University School of Medicine, Shanghai, 200011 China

**Keywords:** Osteoporosis, Bone turnover, Osteoprotegerin, RANKL, Meta-analysis

## Abstract

**Objectives:**

The OPG/RANKL signal pathway was important regulation mechanism of bone remodeling cycle, but the effect of osteoprotegerin (OPG) and RANKL in osteoporosis was uncertain. We did a systematic review with meta-analysis to assess the association between serum OPG/RANKL and osteoporosis.

**Methods:**

The systematic search, data extraction, critical appraisal, and meta-analysis were performed according to the Preferred Reporting Items for Systematic Reviews and Meta-Analyses (PRISMA) statement. Randomized controlled studies were searched in PubMed, OvidMedline, Embase (1946 to present). Standard mean difference (SMD), and associated credible interval (CI) were calculated using RevMan statistical software to assess the continuous data. Heterogeneity in studies was measured by *I*^2^ values. Subgroup analysis was performed based on different bone turnover.

**Results:**

A total of 5 randomized controlled studies met the inclusion criteria. Both OPG and RANKL had no significant differences between the osteoporosis and control group, and the statistical heterogeneity was high in meta-analysis. However, RANKL had significant differences between the osteoporosis group with low bone turnover and control group (SMD =  − 1.17; 95% CI − 1.77 to 0.57; *P* value < 0.01) in subanalysis. Furthermore, the OPG/RANKL ratio was significant lower in the osteoporosis group than in the control group (SMD =  − 0.29; 95% CI − 0.57 to − 0.02; *P* value < 0.05), and the statistical heterogeneity was very low (Chi^2^ = 0.20, *P* = 0.66, *I*^2^ = 0%).

**Conclusions:**

Our meta-analysis study supported OPG and RANKL were important modulatory factors of bone formation and resorption in bone turnover, respectively. Although the serum level of both OPG and RANKL were not associated with osteoporosis, but the OPG/RANKL ratio was associated with osteoporosis. In future, standardizing the test method and unit was good to clinical application.

## Introduction

In healthy adults the bone remodeling cycle displays tight coupling between bone resorption and bone formation. Osteoporosis is the most common metabolic bone disorder and resultant fragility fractures are associated with increased morbidity and mortality [1]. Osteoporosis may be a consequence of (i) a failure to reach normal peak bone mass during growth (ii) a relative increase in bone resorption during adulthood or (iii) a relative reduction in bone formation during adulthood. Whilst osteoporosis has many and diverse causes, uncoupling of the bone remodeling cycle and increased bone resorption relative to formation is a common underlying pathophysiological mechanism. Identification of the RANKL/RANK/OPG Signaling Pathway in the 1990s was a crucial breakthrough in understanding the regulation of osteoclastogenesis in the remodeling cycle. Receptor activator for nuclear factor B ligand (RANKL) binding to its receptor, RANK, on osteoclastic precursor cells, drives further osteoclast differentiation and facilitates fusion, activation and survival [2]. Osteoprotegerin (OPG), a decoy receptor for RANKL, was identified prior to the discovery of RANK/RANKL. It is secreted by osteoblasts and osteocytes and is able to inhibit osteoclastic bone resorption by binding to RANKL and preventing its binding to RANK [3]. Thus, the OPG/RANKL ratio is key in the regulation of bone resorption, bone mass and skeletal integrity [2]. Several studies have assessed the clinical importance of serum concentration of OPG and latterly of serum RANKL in relation to postmenopausal osteoporosis. Low serum OPG has been associated with prevalent vertebral fracture in one study of osteoporotic postmenopausal women [4]. And the high serum RANKL was related to osteoporosis and bone resorption [5, 6]. Because bone resorption is regulated by the relative expression and production of OPG and RANKL levels, the OPG/RANKL ratio has been shown to have a central role in bone resorption in postmenopausal osteoporotic women [7].

However, others’ results were paradoxical. It was observed that an increase in serum OPG correlated negatively with body mass index and/or bone mineral density (BMD) [8]. Yano et al. showed that serum OPG was significantly higher in osteoporotic women compared with age-matched controls. This finding has been confirmed in subsequent studies [9, 10]. Furthermore, Schettet al. showed that low levels of serum RANKL and high levels of serum OPG were associated with incidence of nontraumatic fracture [11]. Herein, we conducted an update meta-analysis to comprehensively assess the association between serum OPG/RANKL and osteoporosis, which provides a clinically useful summary that can guide biomarker selection in research and evaluation of osteoporosis [12].

## Materials and methods

### Searches

**Fig. 1:**
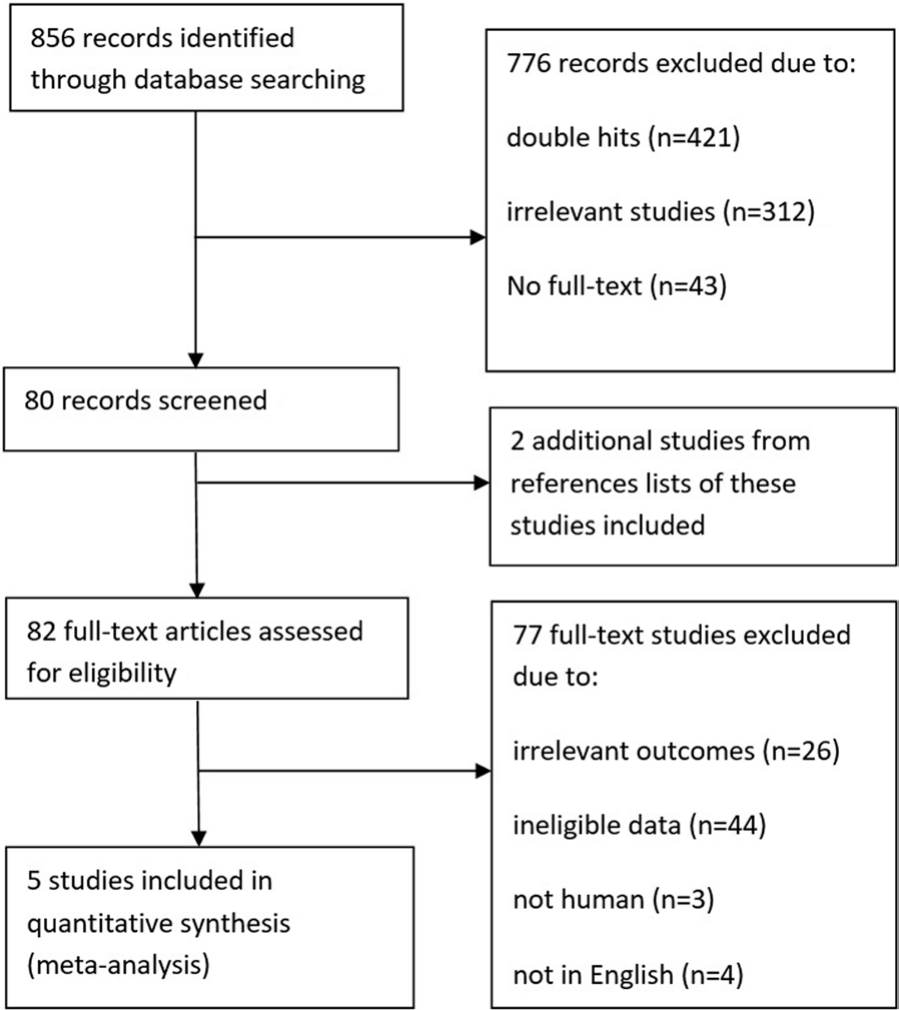
Study flow chart. A total of 433 studies were obtained with the search strategy of which 5 were included in the systematic review and meta-analysis

The search was conducted in accordance with the PRISMA (Preferred Reporting Items for Systematic Reviews and Meta-Analyses) recommendations [13]. We searched PubMed, OvidMedline, Embase and the trial databases of the main regulatory agencies to identify relevant studies published between Jan 1, 1946, and Jun 1, 2022. Figure 1 shows full details of the review methods and the search strategy. The keywords (in English) “osteoprotegerin or RANKL”, “osteoporosis or bone remodeling or bone turnover”, “randomized controlled trial” and “human” were used. Additionally, the reference sections of review articles that were found using this search strategy were screened for possibly suitable original.

### Articles inclusion and exclusion criteria

We included all cohort trials comparing patients with osteoporosis to control. The osteoporosis was showed by significantly lower BMD, lower bone formation or higher bone resorption compared to control. BMD was expressed in absolute values (g/cm2), as well as Z-scores and T-scores (deviation from the peak BMD), which represents the number of standard deviations from a young, sex- and ethnic group-specific reference mean. According to the World Health Organization’s (WHO) definitions, T scores were used as the basis for diagnosis as follows: normal bone mineral density, T score greater than-1; osteopenia, T score less than or equal to − 1 but greater than − 2.5; and osteoporosis, T score less than or equal to − 2.5. For biochemical assay peripheral blood was taken in the morning hours. Serum or plasma concentrations of bone metabolism markers were determined (essentially as described by the manufacturer) by immunoenzymatic ELISA assay. As for the studies, we only selected original full text articles investigating BMD or bone remodeling or bone turnover markers related to etiology or diagnosis of osteoporosis, and excluded the following articles that appeared upon the search strategy detailed above: double hits, reviews, letters to the editor, papers investigating other disorders, articles in non-English and all articles that did not evaluate osteoporosis. First, the titles and abstracts of all articles found were screened for suitability; the initially chosen articles were then screened again checking the entire article.

### Data extraction

The data extraction was carried out by the members of our review team (GC, LQ and JM). Concerning the updated search, all reviewers independently reviewed references and abstracts. And the authors independently screened the titles and abstracts of potentially eligible articles. The full-text of the selected studies was examined. If all reviewers agreed that the trial did not meet eligibility criteria, we excluded it. We obtained the full text of all remaining articles and used the same eligibility criteria to determine which, if any, to exclude at this stage. Any member’s disagreements were solved via discussion with another reviewer (All authors cross-checked the extraction forms for correctness). The same reviewers independently read each article, assessed the completeness of the data abstraction, and confirmed the quality rating. Information extracted included study characteristic (such as lead author and publication year), way of evaluating osteoporosis (such as BMD or bone remodeling or bone turnover markers), and outcome measures (OPG, RANKL or the OPG/RANKL ratio). Data were categorized according to the difference of investigated targets and bone turnover, and meta-analyzed finally.

### Data synthesis and statistical analysis

We produced descriptive statistics for study population characteristics across all eligible trials, describing the number of participants and subgroup, and change of OPG, RANKL or the OPG/RANKL ratio in the osteoporosis and control group. According to study protocol, only cohort studies comparing same bone turnover markers (OPG, RANKL or the OPG/RANKL ratio) were included in the meta-analysis of biomarkers with at least two studies using RevMan analysis software (RevMan 5) of the Cochrane Collaboration [14]. To keep studies comparable, we converted RANKL or OPG pg/mL to pmol/lite by multiply by 500 [5]. At first we did the pair-wise meta-analyses by synthesizing studies that compared the same marker in a random-effects model to incorporate the assumption that the different studies could estimate different, yet related, treatment effects [15, 16]. Subgroup meta-analysis was made according to high bone turnover (the experimental group was comparatively high bone resorption) or low bone turnover (the experimental group was comparatively low bone formation) [2]. For every comparison between osteoporosis and control groups, the standardized mean difference (Hedges’ adjusted SMD) was calculated as the effect size for continuous outcomes with a 95% credible interval (CI). Then outcomes of heterogeneity (shown by the value of *I*^2^) of relevant studies were analyzed too, and we used a p value from a standard test for heterogeneity to further assess coherence of results from different studies in evaluating osteoporosis. *I*^2^ statistics wherein less than 30% was considered to have low heterogeneity. When p value was less than 0.05, the heterogeneity was thought significant. Finally, we screened the efficacy of OPG, RANKL and the OPG/RANKL ratio in evaluating osteoporosis and presented the results in order by comprehensively analyzed SMD and *I*^2^ of each biomarker.

## Results

### Study selection

For the number of found, selected, excluded and finally included articles see the algorithm shown in Fig. 1. Literature search was through English PubMed, OvidMedline and Embase between 1946 and 2021. The initial literature search retrieved the following numbers of articles: PubMed (153-70), OvidMedline (367) and Embase (336).The total number of article is 856 and 421 of them are duplicates. The left 435 were screened and further 355 excluded after initial screening of titles and abstracts (312) and without full-text (43). During these time 2 additional studies from references lists of these studies included. Then, 82 full-text articles were assessed for eligibility with 5 studies included and 77 original articles excluded. Among these excluded ones, there’s 26 with irrelevant outcomes, 44 articles without reliable data (not given in the article or could not be calculated), 3 not human studies and 4 articles in non-English. Finally, 5 studies were suitable for systematic review and meta-analysis. The flow diagram with detailed information was outlined in Fig. 1. Table 1 summarizes key information of the 5 selected articles [5, 17–20] that evaluate osteoporosis by OPG, RANKL or the OPG/RANKL ratio. In this study, the osteoporosis group showed significantly lower BMD (40%, 2/5 studies), high bone turnover (40%, 2/5 studies), or lower bone turnover (20%, 1/5 studies). Eighty percent of studies investigated OPG and RANKL, and 40% studies investigated OPG/RANKL ratio (Table 1).

**Table 1:** Characteristics of 5 included studies

References	Characteristics of experiment group	Outcomes of markers
Anastasilakis [17]	High bone resorption; bone formation not change	RANKL↑
Buxton [5]	Low bone resorption; lower bone formation	OPG; RANKL↓
Gaffney [18]	Low BMD	OPG; RANKL; OPG/RANKL ratio↓
Messalli [19]	High bone resorption; BMD not high	OPG↓
Marques [20]	Low BMD	OPG; RANKL; RANKL/OPG ratio

Up-arrow showed the marker was significantly higher in the osteoporosis group than in the control group. Down-arrow showed the marker was significantly lower in the osteoporosis group than in the control group. No arrow showed there’s no significant difference for the marker between the osteoporosis and control group

*OPG* osteoprotegerin, *RANKL* receptor activator of the nuclear factor B ligand, *BMD* bone mineral density

### Quality assessment

Two authors (GC and JM) discussed the risks of bias in all the included studies as being low risk, unclear, and high risk. A third reviewer (WW) arbitrated unresolved disagreements. One article reported methods regarding randomization sequence generation and allocation concealment [20], two studies [19, 20] performed blinding both of participant, personnel and outcome assessment. Other studies presented unclear risk as they did not show the method of generating randomization and allocation concealment, performed blinding both of participant, personnel and outcome assessment, or reported incomplete outcome data and reporting bias. Thus, corresponding domain was assessed as “low risk”, and no other bias sources were assessed in this meta-analysis.

### Meta-analysis on OPG

**Fig. 2:**
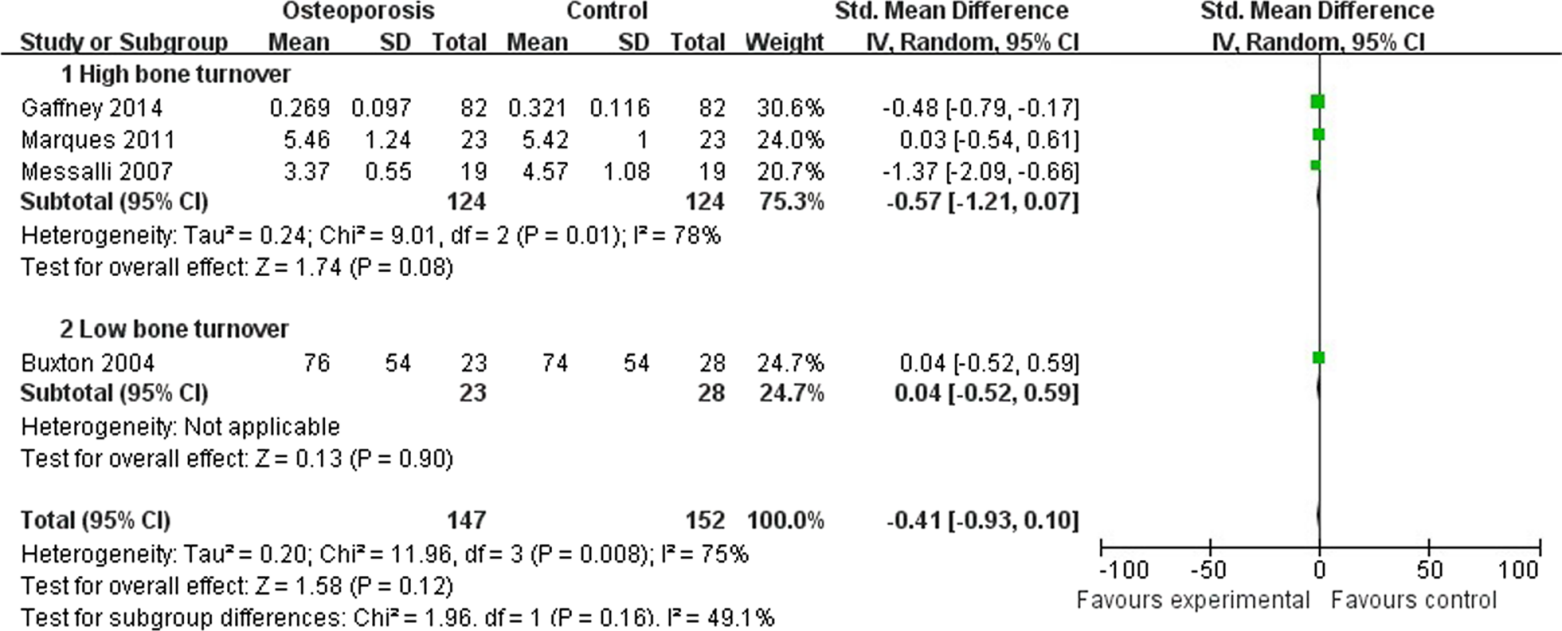
Forest plot on OPG comparison between osteoporosis including both high and low bone turnover and control group

A total of 4 studies that included 152 patients with osteoporosis compared 147 control persons investigated the relationship between OPG and osteoporosis. One study showed the level of serum OPG was significantly lower in the osteoporosis group than in the control group [19]. However, the synthetic results showed there’re no significant differences between the osteoporosis and control group (SMD = −0.41; 95% CI − 0.93 to 0.10; *P* value = 0.12), and the statistical heterogeneity was identified (Chi^2^ = 11.96, *P* < 0.01, *I*^2^ = 75%). As for subgroup analysis, there’re still no significant differences between the osteoporosis group with high bone turnover and control group (SMD = 0.57; 95% CI − 1.21 to 0.07; *P* value = 0.08), and the statistical heterogeneity had significant differences (Chi^2^ = 9.01, *P* = 0.01, *I*^2^ = 78%) [18–20]. Furthermore, there’re still no significant differences between the osteoporosis group with low bone turnover and control group (SMD = 0.04; 95% CI − 0.52 to 0.59; *P* value = 0.90) [5]. And the test for subgroup differences was also high (Chi^2^ = 1.96, *P* value = 0.16, *I*^2^ = 49.1%) (Fig. 2). These results of meta-analysis showed OPG had no association with osteoporosis.

### Meta-analysis on RANKL

**Fig. 3:**
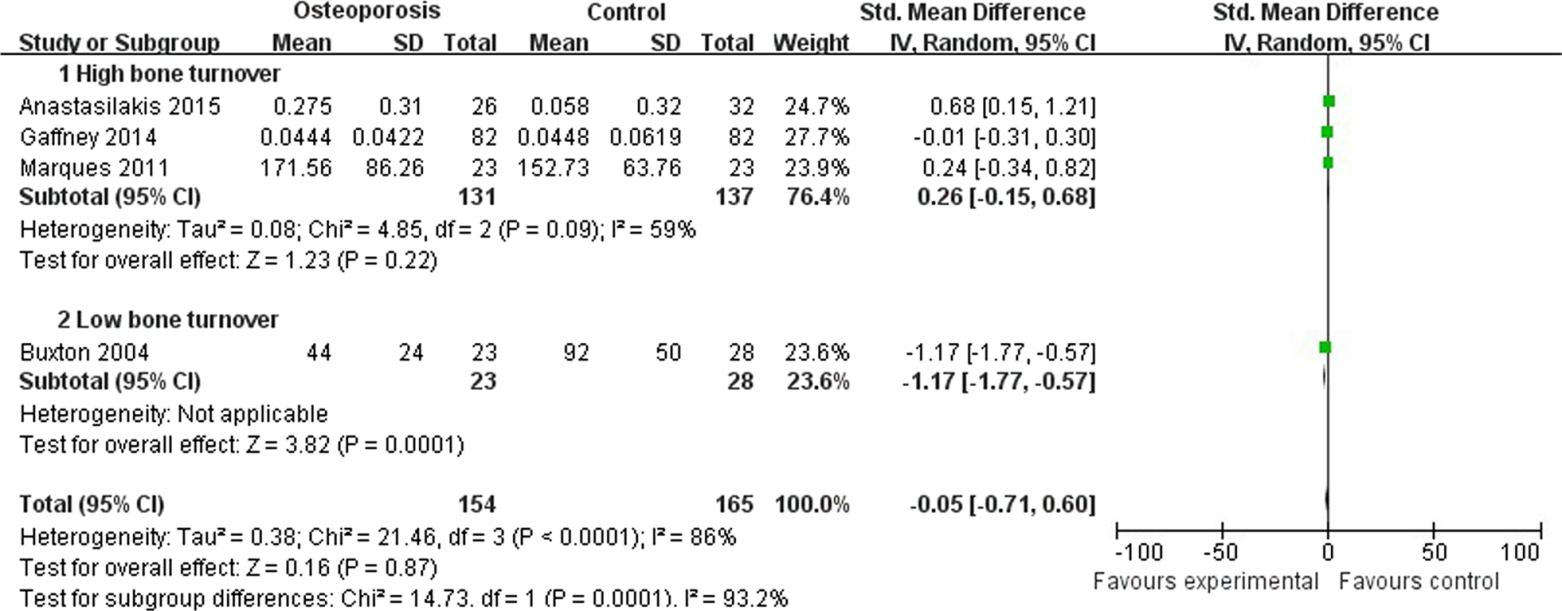
Forest plot on RANKL comparison between osteoporosis including both high and low bone turnover and control group

A total of 4 studies that included 154 patients with osteoporosis compared 165 control persons investigated the relationship between RANKL and osteoporosis. The synthetic results showed there’re no significant differences between the osteoporosis and control group (SMD =  − 0.05; 95% CI − 0.71 to 0.60; *P* value = 0.87), and the statistical heterogeneity was significant (Chi^2^ = 21.46, *P* < 0.01, *I*^2^ = 86%). As for subgroup analysis, there’re still no significant differences between the osteoporosis group with high bone turnover and control group (SMD = 0.26; 95% CI − 0.15 to 0.68; *P* value = 0.22), and the statistical heterogeneity was also high (Chi^2^ = 4.85, *P* = 0.09, *I*^2^ = 59%) [17, 18, 20]. However, there’re significant differences between the osteoporosis group with low bone turnover and control group (SMD = −1.17; 95% CI − 1.77 to 0.57; *P* value < 0.01) [5]. And the test for subgroup heterogeneity differences was also significant (Chi^2^ = 14.73, *P* value < 0.01, *I*^2^ = 93.2%) (Fig. 3). These results of meta-analysis showed RANKL was not associated with osteoporosis, but may be associated with bone resorption.

### Meta-analysis on OPG/RANKL ratio

**Fig. 4:**
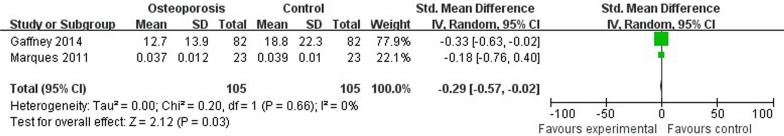
Forest plot on OPG/RANKL ratio comparison between osteoporosis and control

A total of 2 studies that included 105 patients with osteoporosis compared 105 control persons investigated the association of the OPG/RANKL ratio with osteoporosis. One study showed the OPG/RANKL ratio had no significant differences between the osteoporosis and control group [18]. However, the synthetic results showed the OPG/RANKL ratio was significant lower in the osteoporosis group than in the control group (SMD = −0.29; 95% CI − 0.57 to − 0.02; *P* value < 0.05), and the statistical heterogeneity was very low (Chi^2^ = 0.20, *P* = 0.66, *I*^2^ = 0%) (Fig. 4). These results of meta-analysis showed the OPG/RANKL ratio was associated with osteoporosis.

## Discussion

In the field of bone biology have, for a long time, sought to understand the mechanisms responsible for the cross-talk between osteoblasts and osteoclasts. A major step toward answering this question was provided by the discovery of OPG, the decoy receptor for RANKL [10]. It’s recently reported the denosumab, a human monoclonal IgG2 antibody that binds RANKL and thus inhibits its activity, is the most potent antiresorptives, as reflected by its ability to reduce the bone resorption marker C-telopeptide of type I collagen (CTX), and increase BMD [21–23] However, as we previously introduced, the effect of OPG, RANKL and even OPG/RANKL ratio [20, 24] in osteoporosis was controversial. Our meta-analysis results showed the serum level of OPG had no association with osteoporosis including both high and low bone turnover and the statistical heterogeneity was significant. Osteoporosis usually develop slowly, and bone turnover markers was more sensitive and changed earlier than BMD [25, 26], so the serum OPG level was able to be elevated in osteoporosis, which is considered as a compensation for the persisted bone loss after menopause in osteoporotic women [27]. In high bone turnover OPG was able to decrease [19], while in low bone turnover OPG may increase to rebalance bone turnover [8]. Therefore, the serum level of OPG was not related to osteoporosis or bone turnover, but may be related to bone formation.

As for RANKL, our meta-analysis results showed that the serum level of RANKL had no association with osteoporosis including high bone turnover and the statistical heterogeneity was high. However, the serum RANKL level was significantly lower in patients with osteoporosis with low bone turnover. Our results supported that RANKL was antagonistic factor of OPG and the modulatory factor of bone resorption, because RANKL was able to increase in high bone turnover [17], while RANKL decrease in low bone turnover. Therefore, it’s why RANKL was not related to osteoporosis.

Most importantly, although there’s study [18] that showed OPG/RANKL ratio had no significant differences between the osteoporosis and control group, our meta-analysis results showed the ratio of serum level of OPG and RANKL was significantly lower in patients with osteoporosis. Furthermore, the statistical heterogeneity was very low. Therefore, our meta-analysis results showed OPG and RANKL surely was important modulatory factors of osteoporosis, and yet it’s not single OPG or RANKL, but OPG/RANKL ratio was associated with osteoporosis. In our meta-analysis, we found there’re many other reasons that leads to controversial conclusions for the effect of OPG and RANKL in osteoporosis. First, the long term RCT was few due to difficulties and large investment of investigation. Second, examination methods were various and even the test units had huge differences. Finally, the studies with large population were still very few.

In conclusion, our meta-analysis study supported OPG and RANKL were important modulatory factors of bone formation and resorption in bone turnover, respectively. Although the serum level of both OPG and RANKL were not related with osteoporosis, but OPG/RANKL ratio was associated with osteoporosis. In future, it still need more long term and larger sample RCT or multi-center studies to reassure the effect of OPG and RANKL in osteoporosis. Furthermore, it also need standardizing the test method and unit to make it available clinical application as useful bone remodeling biomarkers.

## Data Availability

The data that support the findings of this study are available on request from the corresponding author.
